# Evaluating the readability, quality, and reliability of responses generated by ChatGPT, Gemini, and Perplexity on the most commonly asked questions about Ankylosing spondylitis

**DOI:** 10.1371/journal.pone.0326351

**Published:** 2025-06-18

**Authors:** Mete Kara, Erkan Ozduran, Müge Mercan Kara, İlhan Celil Özbek, Volkan Hancı

**Affiliations:** 1 Izmir City Hospital, Internal Medicine, Rheumatology, Izmir, Turkey; 2 Sivas Numune Hospital, Physical Medicine and Rehabilitation, Pain Medicine, Sivas, Turkey; 3 Izmir City Hospital, Neurology, Pain Medicine, Izmir, Turkey; 4 University of Health Sciences, Derince Training and Research Hospital, Physical Medicine and Rehabilitation, Kocaeli, Turkey; 5 Dokuz Eylul University, Anesthesiology and Reanimation, Critical Care Medicine, Izmir, Turkey; The University of Texas Health Science Center at Houston / McGovern Medical School, UNITED STATES OF AMERICA

## Abstract

Ankylosing spondylitis (AS), which usually occurs in the second and third decades of life, is associated with chronic pain, limitation of mobility, and severe decreases in quality of life. This study aimed to make a comparative evaluation in terms of the readability, information accuracy and quality of the answers given by artificial intelligence (AI)-based chatbots such as ChatGPT, Perplexity and Gemini, which have become popular with the widespread access to medical information, to user questions about AS, a chronic inflammatory joint disease. In this study, the 25 most frequently queried keywords related to AS determined through Google Trends were directed to each 3 AI-based chatbots. The readability of the resulting responses was evaluated using readability indices such as Simple Gunning Fog (GFOG), Flesch Reading Ease Score (FRES) and Measure of Gobbledygook (SMOG). The quality of the responses was measured by Ensuring Quality Information for Patients (EQIP) and Global Quality Score (GQS) scores, and the reliability was measured using the modified DISCERN and Journal of American Medical Association (JAMA) scales. According to Google Trends data, the most frequently searched keywords related to AS are “Ankylosing spondylitis pain”, “Ankylosing spondylitis symptoms” and “Ankylosing spondylitis disease”, respectively. It was found that the readability levels of the answers produced by AI-based chatbots were above the 6th grade level and showed a statistically significant difference (p < 0.001). In EQIP, JAMA, mDISCERN and GQS evaluations, Perplexity stood out in terms of information quality and reliability, receiving higher scores compared to other chat robots (p < 0.05). It has been found that the answers given by AI chatbots to AS-related questions exceed the recommended readability level and the reliability and quality assessment raises concerns due to some low scores. It is possible for future AI chatbots to have sufficient quality, reliability and appropriate readability levels with an audit mechanism in place.

## Introduction

Ankylosing spondylitis (AS) is a chronic and progressive inflammatory disease primarily involving the axial skeleton [1]. AS, the most common form of axial spondyloarthropathies (SpA), is characterised by inflammation of the spine and sacroiliac joints. Over time, this inflammation can lead to decreased spinal mobility and, in severe cases, spinal fusion [2]. AS, which usually occurs in the second and third decades of life, is associated with chronic pain, limitation of mobility, and severe decreases in quality of life. Genetic predisposition, especially HLA-B27 positivity, is known to play an important role in the development of AS [3]. While the diagnostic process is based on the patient’s symptoms, critical diagnostic tools such as magnetic resonance imaging (MRI) and HLA-B27 testing can be used [4]. The global prevalence of AS ranges from approximately 0.1% to 1.4% [4].

According to the axial spondyloarthritis management recommendations prepared by The Assessment of SpondyloArthritis international Society (ASAS)-EULAR in 2022, it is important to inform the patient correctly about his disease and encourage him to be educated, exercise and quit smoking [5]. It is strongly emphasized that all patients should receive education about the disease as a starting point for self-management, to engage and empower them as active partners in their care [6]. Despite all these positive recommendations, the healthcare provider’s knowledge and communication skills, as well as the ability to assess patients’ educational needs, can impact patient education [7]. It is reported that lack of information about health care provider causes delays of 7–10 years in the diagnosis process [8]. Not only this, but also the patient’s social and cultural background and physiological factors may also create some barriers. Finally, the level of health literacy that helps patients access, understand, evaluate, and apply information regarding healthcare also plays a major role [7].

Accessing online information in the field of health is not a new issue. According to a study, 9 out of 10 Americans accessed the internet in 2018, and 75% of them tried to access information in the field of health [9]. With the release of version 3.5 of the Chat Generative PreTrained Transformer (ChatGPT) in 2022, a large language model (LLM) powered by AI, a new alternative search engine has emerged instead of traditional internet search engines. Perplexity AI (2022) and Google Gemini (2024- formerly BARD 2023) were other applications that entered the AI market as “chatbots” that use natural language processing (NLP) and machine learning to answer user questions [10]. It is estimated that ChatGPT has 300 million weekly active users and 3.8 billion monthly visitors according to November 2024 data [11]. Many AI applications, especially the AI intelligence applications listed above, have been the subject of studies on different medical subjects in which the answers given by users to their questions are evaluated [12,13].

A popular topic in studies created with AI applications is rheumatological diseases. [14]. Many benefits are mentioned in the literature that AI applications can facilitate screening, diagnosis, monitoring, risk assessment, determination of prognosis, obtaining optimal treatment results and new drug discovery for patients with rheumatoid arthritis [15]. According to the study, it is reported that 58.9% of the patients diagnosed with rheumatoid arthritis, AS and fibromyalgia have access to information about their diseases via the internet [16]. It is well known that; patients who are informed about the causes, pathophysiology, treatment and prevention of a disease can better participate and adapt to disease prevention or treatment procedures [9]. The contribution of data provided by AI applications to the field of health in different rheumatological diseases such as AS can be discussed in future studies and can help this field.

According to studies, it is stated that information and communication technology-based patient education has the potential to improve self-management and behavioral changes in patients in autoimmune rheumatological diseases [17]. The use of AI in rheumatic diseases has increased the diagnosis and accuracy of rheumatic diseases, made it possible to predict patient outcomes, expanded treatment options, and facilitated the provision of personalized medical solutions [18]. AI-powered tools facilitate personalized patient education and provide remote diagnostic and treatment support [19]. It can be stated that AI-supported applications have promising potential in the future in early diagnosis and appropriate treatment planning for the patient in chronic inflammatory and multisystemic diseases such as AS, as in other rheumatic diseases. However, despite all these positive effects, it is of great importance that patients have access to reliable and understandable sources when searching for information online about chronic diseases such as AS. Misinformation can cause unnecessary anxiety and treatment non-compliance [14].

Readability is defined as a criterion that determines the comprehensibility of written materials by the target audience. Readability level can be determined by formulae such as Flesch-Kincaid, Gunning Fog and SMOG. The readability level for patient education materials (PEM) is recommended by the American Medical Association (AMA), the National Institutes of Health (NIH), and the US Department of Health and Human Services to be grade 6 or lower. This standard aims to ensure that patients have access to accurate and understandable information about their disease and treatment options [20,21].

Texts that are difficult to read negatively affect health literacy and prevent patients from managing chronic diseases. Patients have difficulty even following the medication instructions they should use [22]. On the contrary, high health literacy resulting from more easily readable texts is associated with appropriate medication adherence [23].

This study aims to contribute to the literature by comprehensively evaluating the reliability, quality and readability of health information about AS produced by three AI – supported chatbots: ChatGPT, Perplexity and Gemini. The lack of a studies in the literature analyzing the texts produced by AI-assisted chatbots about AS increases the importance of this research. Patients’ access to more reliable and understandable information will contribute to more favorable outcomes in disease management.

## Materials and methods

### Ethics comittee permission

The planning, execution, and data collection processes of this cross-sectional study were carried out in accordance with the approval of the relevant ethics committee. (Cumhuriyet University Ethics Committee, Ethics Committee No: 2024-10/10, Date: 17.10.2024). Written or verbal informed consent was not obtained because the current study did not include human participants or human tissue.

In this study, similar to previous studies in the literature, the answers given by AI chatbots to the most frequently asked questions about AS were evaluated in a cross-sectional manner within a static time period [13]. For this reason, it is clear that different study results may occur in different time periods.

### Research procedure

At the beginning of the study, personal internet browser data was completely deleted and Google Incognito mode was activated in order to eliminate possible biases. However, in real life, although the algorithmic biases are mitigated by using Google Incognito mode, disabling the user’s lifestyle and searches may lead to different results, which may cause a limitation [24].

On the Google Trends (https://trends.google.com/) platform, the frequencies and geographical distributions of searches made on the keyword “Ankylosing Spondylitis” worldwide from 2004 to the present were examined on October 18, 2024, using the “most relevant” results filter and The 25 most frequently searched keywords and geographical areas of interest were identified and recorded [9]. Measurements made in January 2024 showed that Google’s share in the search engine market was 81.95%. Considering this overwhelming superiority, the Google search engine was used in our study to access the most reliable and largest database [25]. In our study, the 25 most frequently asked questions on Google Trends were evaluated [26]. Just as there are studies similar to this methodology in the literature, there are also studies in the methodology in which the 100 most frequently asked questions about any medical subject are questioned. The 100 questions obtained in these studies were evaluated separately by asking AI chatbots one by one and a larger sample was obtained [20,21]. In addition, there are AI studies that examine the most frequently asked questions on the official websites of some communities and organizations [27]. Although studies with different methodologies change the comprehensiveness of the studies by creating sample size differences in the sample, they add their own uniqueness to each study.

The study aimed to examine the responses of freely available AI models, including ChatGPT, Gemini and Perplexity, to the specified keywords. Accordingly, the obtained keywords were directed to AI models in English [20,28]. AI Chatbots have different working principles. Generative Pre-trained Transformer (GPT) is a natural language generation model developed by OpenAI. It learns to predict the next word in a given text and produces meaningful text that resembles the typed content [29]. Perplexity; It is a chatbot based on OpenAI GPT technology and provides answers containing references to queries and directions. Gemini AI is a “native multimodal” model that allows it to process and learn from various types of data, including text, video and audio. Gemini is capable of analyzing complex data sets such as images and subgraphs [12].

It is known that underlying machine learning methods are not well positioned to distinguish between factually correct and incorrect information during data entry, which is why AI applications regularly make factual errors and provide imprecise information [30]. Cyber crimes may occur as a result of entering personal information being recorded by chatbots, creating fake profiles and manipulating images. This situation brings with it some concerns about loss of privacy about our own data [31]. In the light of this informations, different user logins were made and each keyword was asked to the AI chatbots, thus trying to prevent biases that may occur during the sequential processing of keywords. The answers produced by the AI models were recorded in the database to be evaluated with readability, reliability and quality metrics. The keywords and responses from each AI chatbot can be accessed through the web archive found at: https://figshare.com/articles/dataset/AI-AS_Responds_docx/28395005?file=52285787

In this study, the free and publicly available GPT-4o model of the ChatGPT free version, which has the highest level of social accessibility of AI technologies, was used. In this way, it is aimed to evaluate the information about the content presented to individuals at different socioeconomic levels when they benefit from these Technologies [12,20].

### Readability evaluation

#### Readability formulas.

The responses of AI chatbots to the keywords they produced were examined with various formulas using the website that measures text readability (http://readabilityformulas.com/). Since there is no information about which readability index provides more accurate and reliable information or a gold standard readability index, the most popular indexes used in previous studies, similar to the literature, were used in our study.

In this study, the Coleman-Liau Readability Index (CLI), Linsear Write (LW), Automated Readability Index (ARI), Simple Measure of Gobbledygook (SMOG), Gunning Fog Readability Index (GFOG), The Flesch Reading Ease Score (FRES) and Commonly used readability metrics such as Flesch-Kincaid Grade Level (FKGL) were used. These metrics were used to determine how close the texts produced by AI are to daily spoken language and their level of comprehensibility [20,21,32].

#### Readability score evaluation.

Detailed information on how different readability formulas work is presented in Table 1. The obtained readability scores are expressed as median (minimum-maximum) values to determine the general readability level of the texts. In this study, the results obtained were compared with the sixth grade readability level recommended by the American Medical Association and the National Institutes of Health (NIH). Accordingly, the average acceptable score for the Flesch Reading Ease Score (FRES) was determined as 80.0, and for the other six formulas as 6. Final scores below 80.0 for the FRES formula and above 6 for other formulas represent text above the average recommended readability level [20,21].

**Table 1:** Readability tools, formulas and descriptions.

Readability Index	Description	Formula
**Gunning FOG (GFOG)**	It estimates the number of years of education required for a person to understand a given text.	G = 0.4 X (W/S+((C*/W) X 100))
**Flesch Reading Ease Score(FRES)**	It was created to assess the readability of newspapers and is particularly effective for evaluating school textbooks and technical manuals. The scores range from 0 to 100, with higher scores indicating greater ease of reading.	I = (206.835 – (84.6 X (B/W)) – (1.015 X (W/S)))
**Flesch–Kincaid grade level (FKGL)**	Delineates the academic capacity level imperative for grasping the written material	G = (11.8 X (B/W)) + (0.39 X (W/S)) −15.59
**Simple Measure of Gobbledygook (SMOG)**	It measures the number of years of education the average person needs to understand a text.	G = 1.0430 X √C + 3.1291
**Coleman–Liau (CL) score**	Evaluates the educational level required for understanding a text and offers an associated grade level in the US education system.	G = (−27.4004 X (E/100)) + 23.06395
**Linsear Write (LW)**	Offers an approximate assessment of the academic level needed to comprehend the text.	LW = (R + 3C)/S Result • If >20, divide by 2 • If ≤20, subtract 2, and then divide by 2
**Automated readability index (ARI)**	Assesses the scholastic rank in American educational institutions needed to be capable of comprehending written material. The greater the number of characters, the more complex the term.	ARI = 4.71 X l + 0.5*ASL – 21.43

G = Grade level; B = Number of syllables; W = Number of words; S = Number of sentences; I = Flesch Index Score; SMOG = Simple Measure of Gobbledygook; C = Complex words (≥3 syllables); E = predicted Cloze percentage = 141.8401 – (0.214590 X number of characters) + (1.079812*S); C* = Complex words with exceptions including, proper nouns, words made 3 syllables by addition of “ed” or “es”, compound words made of simpler words. ASL = the average number of sentences per 100 words R = the number of words ≤2 syllables.

### Reliability evaluation

In our study, the Modified DISCERN scale was used to determine the reliability level of information sources. This scale evaluates five different criteria and gives sources a score between 0 and 5. A higher score means that the source provides information with higher reliability [33].

The questions in the scale evaluate five basic dimensions such as the timeliness of the sources, whether additional sources of information are listed, whether they address discussions, and the clarity and impartiality of the language [34]. The validity and reliability of the JAMA and DISCERN scales have been tested in previous studies [35,36].

In the analysis carried out based on “The Journal of the American Medical Association (JAMA) Benchmark”, which is another reliability scale we used in our study, the scientific reliability of the texts was evaluated within the framework of four basic publication ethics principles such as authorship, currency, disclosure and attribution.

In the evaluation made according to JAMA criteria, 0 or 1 point is given for each criterion depending on whether the study meets this criterion or not. The total score obtained in this way indicates the overall reliability level of the study between 0 and 4. Higher scores indicate that the study meets these criteria better and is therefore more reliable [14,37].

### Quality evaluation

The Global Quality Score (GQS) is a scale that evaluates the quality of online health information on a five-point scale. On this scale, a score of 1 represents the lowest quality and a score of 5 represents the highest quality. Accordingly, a health source with a score of 1 does not provide any benefit to patients, while a source with a score of 5 provides extremely reliable and comprehensive information. Likewise, 2 points are considered low quality and limited in use, 3 points are considered medium quality and limited useful, and 4 points are considered good quality and useful [38–40]. The reliability and validity of the GQS questionnaire are found in the literature [41].

EQIP (Ensuring Quality Information for Patients) is a tool used to evaluate the quality of medical texts. Based on the “yes”, “partially” or “no” answers given to the 20 questions in this tool, the quality of the text is determined with a score between 0 and 100. Scoring is done by giving 1 point for each “yes” answer, 0.5 for the “partially” answer and 0 point for the “no” answer, and the final score is calculated. The final score is divided by 20 and then those that do not apply are subtracted and multiplied by 100 ((X of Yes*1) + (Y of Partly*0.5) + (Z of No*0))/20 – (Q of does not apply))] *100 = % score) [42]. The results are evaluated in four categories, “severe problems with quality” (0−25%), “severe problems with quality” (26−50%), “good quality with minor problems” (51−75%) and “well written” (76−100%) [43].

In our study, the surveys used in AI studies in the literature were carefully evaluated and more than one reliability and quality surveys were included in our study. In the literature, the method of evaluating accuracy on a medical subject through the joint opinion of experienced authors on that subject has not been used because it is not considered objective [44].

### Statistical analysis

We used SPSS Windows version 24.0 (SPSS Inc., USA) to analyze the data. Kolmogorov-Smirnov and Shapiro-Wilk tests were used as normality tests. It was determined that it did not comply with normal distribution. For this reason, nonparametric tests were used. Categorical variables were summarized with frequencies and percentages, while continuous variables were described with medians and their ranges. To compare categorical variables, we employed Fisher’s exact test and the chi-square test. For continuous variables, the Mann-Whitney U and Wilcoxon tests were used. Statistical significance was set at a p-value less than 0.05.

## Results

The most frequently used keywords on Google by users looking for information about ankylosing spondylitis were determined via Google Trends as “Ankylosing spondylitis pain”, “Ankylosing spondylitis symptoms” and “Ankylosing spondylitis disease”. The keyword “Symptoms of Ankylosing spondylitis” was removed from the analysis because the keyword “Ankylosing spondylitis symptoms” was present. The keyword “Treatment for Ankylosing spondylitis” was removed from the analysis because the keyword “Ankylosing spondylitis treatments” existed. The keywords “Arthritis”, “Rheumatoid Arthritis”, “Spondylosis”, “Psoriatic arthritis”, “osteoarthritis”, “as” and “Ankylosing spondylitis Reddit” were removed from the analysis because they were irrelevant to the topic and were answered by AI chatbots by evoking different diseases.

Finally, we focused on the 16 identified keywords. All of these keywords are listed in Table 2. As a result of the analysis, it was determined that the highest search volume regarding AS was in Australia, New Zealand and Ireland, respectively. Data on AS prevalence in Australia is not clear, and it is estimated to be 0.2% in Western Australia. [45]. Promising developments in the treatment of rheumatological diseases in Australia and New Zealand may have increased public awareness in this field and increased online searches [46].

**Table 2:** Top 16 relevant keywords searched about Ankylosing spondylitis across countries: 2004-2023 (Based on Google trends data).

Rank	Keyword
**1**	**Ankylosing spondylitis pain**
**2**	**Ankylosing spondylitis symptoms**
**3**	**Ankylosing spondylitis disease**
**4**	**What is Ankylosing spondylitis**
**5**	**Ankylosing spondylitis treatment**
**6**	**As Ankylosing spondylitis**
**7**	**Ankylosing spondylitis meaning**
**8**	**Ankylosing spondylitis causes**
**9**	**Ankylosing spondylitis diagnosis**
**10**	**Ankylosing spondylitis icd10**
**11**	**Ankylosing spondylosis**
**12**	**Hla b27**
**13**	**Uveitis**
**14**	**Ankylosing spondylitis radiology**
**15**	**Spondyloarthritis**
**16**	**Ankylosing spondylitis blood test**

EQIP: ensuring quality information for patients.

AS-related keywords were entered into ChatGPT-4o, Perplexity and Google Gemini AI chatbots. The readability scores of the answers given by these AI chatbots were calculated. The readability levels of the texts were evaluated by comparing them with the ability of a 6th grade reader to understand the text (Table 3).

**Table 3:** Readability scores for Chatgpt-4o, Gemini, and Perplexity responses to the most frequently asked Ankylosing spondylitis -related questions, and a statistical comparison of the text content to a 6th-grade reading level [Median, 95% Confidence Interval (CI) (Lower limit of confidence interval- Upper limit of confidence interval)].

CALCULATOR Statistics	ChatGPT	Gemini	Perplexity	ChatGPT C6thGRL (P)^*^ ^†^ (Value of Cohen’s d-Effect size)	Gemini C6thGRL (P)^*^ ^†^ (Value of Cohen’s d-Effect size)	Perplexity C6thGRL (p)^*^ ^†^ (Value of Cohen’s d-Effect size)	Between ChatGPT and Gemini (p)^††^	Between ChatGPT and Perplexity (p)^††^	Between Perplexity and Gemini (p)^††^ (Value of Cohen’s d-Effect size)
**FRES**	34.64 (25.05-37.11)	35.04 (30.88-40.7)	21.76 (18.42-28.93)	**< 0.001 (5.2-large)**	**< 0.001 (5.9-large)**	**< 0.001 (6.7-large)**	0.386	0.050	**< 0.001** **(1.27-large)**
**GFOG**	15.35 (14.5-17.09)	13.95 (13.36 −15.34)	16.79 (15.51-17.34)	**< 0.001 (4.02-large)**	**< 0.001 (4.51-large)**	**< 0.001 (6.9-large)**	0.113	0.207	**0.007 (1.16-large)**
**FKGL**	11.87 (11.49-13.85)	11.65 (10.93-12.61)	13.48 (12.75-14.39)	**< 0.001 (3.01-large)**	**< 0.001 (3.7-large)**	**< 0.001 (4.9-large)**	0.498	0.070	**0.003 (1.16-large)**
**CLI**	14.54 (13.9-16)	14.21 (13.31-15.01)	16.40 (14.97 −16.94)	**< 0.001 (4.5-large)**	**< 0.001 (5.1-large)**	**< 0.001 (5.4-large)**	0.336	0.097	**0.024 (1.04-large)**
**SMOG**	11.70 (11.55-13.25)	11.47 (11.05-12.31)	12.96 (12.12-13.71)	**< 0.001 (4.01-large)**	**< 0.001 (4.8-large)**	**< 0.001 (4.6-large)**	0.228	0.309	**0.003 (0.9-large)**
**ARI**	11.94 (11.58-14.07)	11.47 (10.86-12.67)	13.05 (12.52-14.32)	**< 0.001 (2.9-large)**	**< 0.001 (3.4-large)**	**< 0.001 (4.4-large)**	0.228	0.152	**0.024** **(0.98-large)**

Abbreviations: Flesch reading ease score (FRES), Gunning FOG (GFOG), Flesch-Kincaid Grade Level (FKGL), Simple Measure of Gobbledygook (SMOG), Coleman-Liau Index (CLI), Automated Readability Index (ARI) and Linsear Write (LW)

* : C6thGRL**(p):** Comparison of the responses according to 6th grade reading level **(p)**

† : Wilcoxon test

†† : Chi-Square test for categorical variables and Mann-Whitney U test for continuous variables

p *values* in *bold* are *statistically significant*

### Assessment of readability across the three groups, utilizing average scores from the readability calculator

**Fig 1:**
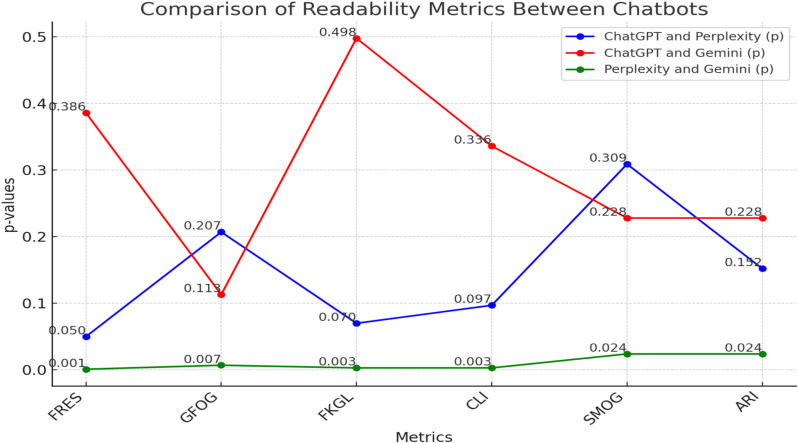
Binary readability evaluation between chatbots.

When evaluating the readability of responses across all three chatbots, significant differences were observed between specific groups. In the readability analysis, no significant difference was found between ChatGPT-4o and Perplexity or ChatGPT-4o and Gemini in pairwise comparisons. Perplexity and Gemini showed significant differences across the FRES, GFOG, FKGL, SMOG, CLI, and ARI readability formulas (p < 0.001 – Cohen’s d = 1.27-large effect, p = 0.007 – Cohen’s d = 1.16 – large effect, p = 0.003 – Cohen’s d = 1.16 – large effect, p = 0.024, p = 0.003 – Cohen’s d = 0.9 – large effect, p = 0.024 Cohen’s d = 0.98 – large effect, respectively). According to the readability evaluations, the metrics rank the systems from easiest to hardest as follows: Google Gemini, ChatGPT-4.0, and Perplexity (Table 3, Fig 1). Although Gemini was the easiest to read compared to other chatbots, it still gave responses that were difficult to read at the recommended sixth-grade level.

### Evaluating the responses from ChatGPT, Gemini, and Perplexity in relation to the recommended sixth-grade reading level

When comparing the median readability scores of all responses to the sixth-grade reading level, a statistically significant difference was found across all metrics (p < 0.001). Notably, the readability of the responses surpassed the sixth-grade standard in every metric (Table 3). People who gain disease-specific knowledge through texts at the recommended readability level can understand the cause and pathomechanics of the disease, have accurate information about prevention and treatment options, and can participate in prevention or rehabilitation more effectively and actively [47].

### Evaluation of reliability and quality

**Fig 2:**
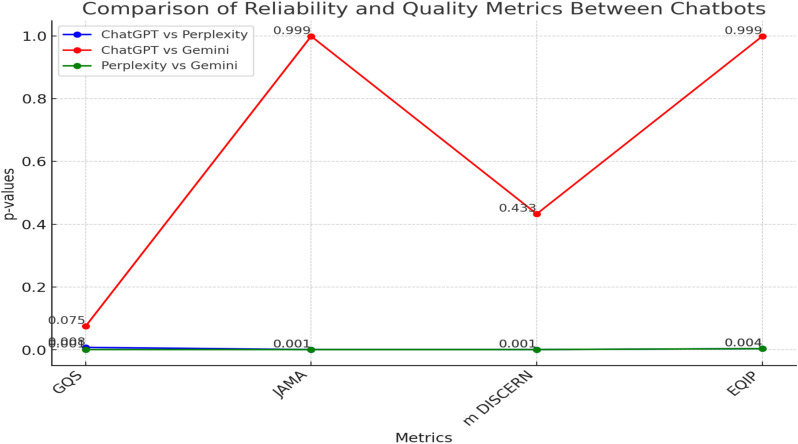
Binary reliability and quality evaluation between chatbots.

The JAMA, DISCERN, GQS and EQIP evaluation results (median, 95% Confidence Interval (CI) (Lower limit of confidence interval- Upper limit of confidence interval)) of the answers given by ChatGPT were as follows: 0 (0–0), 2 (1.6–2.03), 3 (2.5–3.63), 48.50 (45–50.37). The JAMA, DISCERN, GQS and EQIP evaluation results (median, 95% Confidence Interval (CI) (Lower limit of confidence interval- Upper limit of confidence interval))of the answers given by Google Gemini were as follows; 0 (0.16–0.71), 2 (1.36–1.89), 3 (2.63–3.24) and 48.65 (47.58–51.43). The JAMA, DISCERN, GQS and EQIP evaluation results (median, 95% Confidence Interval (CI) (Lower limit of confidence interval- Upper limit of confidence interval)) of the answers given by Perplexity were as follows; 2 (2.16–2.71), 3 (2.97–3.4), 4 (3.8–4.07) and 64.95 (63.13–68.09). Perplexity’s responses obtained the highest Figure scores in EQIP, JAMA, modified DISCERN, and GQS assessments (p < 0.001) (Table 4, Fig 2). The most important factor that could put Perplexity one step ahead of other chatbots may have been the availability of information such as references, author names, and the creation date of the text.

**Table 4:** Comparison of JAMA,EQIP, modified DISCERN and Global Quality Scale (GQS) ratings for the responses from ChatGPT-4o, Gemini, and Perplexity.

	ChatGPT vs Perplexity			ChatGPT vs Gemini			Perplexity vs Gemini		
	**ChatGPT**	**Perplexity**	**P (Value of Cohen’s d-Effect size)**	**ChatGPT**	**Gemini**	**P**	**Perplexity**	**Gemini**	**P (Value of Cohen’s d-Effect size)**
**GQS, n (%)**			**0.008** ^*^ **(1.13 -large)**			0.075^*^			< 0.001^*^ **(2.26-large)**
**1-point**	2(12.5)	0 (0)		2(12.5)	0(0)		0 (0)	0(0)	
**2-point**	2(12.5)	0 (0)		2(12.5)	3(18.7)		0 (0)	3(18.7)	
**3-point**	5(31.3)	1(6.3)		5(31.3)	11(68.8)		1(6.3)	11(68.8)	
**4-point**	7(43,7)	15(93.7)		7(43,7)	2(12.5)		15(93.7)	2(12.5)	
**5-point**	0 (0)	0 (0)		0 (0)	0 (0)		0 (0)	0 (0)	
**JAMA, n (%)**			< 0.001^*^ **(6.73 -large)**			>0.999			< 0.001^*^ **(3.9-large)**
**0-point**	16(100)	0 (0)		16(100)	9(56.3)		0 (0)	9(56.3)	
**1-point**	0 (0)	0 (0)		0 (0)	7(43.7)		0 (0)	7(43.7)	
**2-point**	0 (0)	9(56.3)		0 (0)	0 (0)		9(56.3)	0 (0)	
**3-point**	0 (0)	7(43.7)		0 (0)	0 (0)		7(43.7)	0 (0)	
**4-point**	0 (0)	0 (0)		0 (0)	0 (0)		0 (0)	0 (0)	
**m** **DISCERN, n (%)**			< 0.001^*^ **(3.4 -large)**			0.433**			**< 0.001** ^*^ **(3.-large)**
**1-point**	3(18.8)	0 (0)		3(18.8)	6(37.5)		0 (0)	6(37.5)	
**2-point**	13(81.3)	0 (0)		13(81.3)	10(62.5)		0 (0)	10(62.5)	
**3-point**	0 (0)	13(81.3)		0 (0)	0 (0)		13(81.3)	0 (0)	
**4-point**	0 (0)	3(18.7)		0 (0)	0 (0)		3(18.7)	0 (0)	
**5-point**	0 (0)	0 (0)		0 (0)	0 (0)		0 (0)	0 (0)	
**EQIP, n(%)**			0.004^*^ **(3.7 -large)**			>0.999			0.004^*^ **(3.87-large)**
**Serious problems with qood quality**	8(50)	0 (0)		8(50)	8(50)		0 (0)	8(50)	
**Good quality with minor problems**	8(50)	15 (93.8)		8(50)	8(50)		15 (93.8)	8(50)	
**Well written**	0 (0)	1 (6.2)		0 (0)	0 (0)		1 (6.2)	0 (0)	

EQIP: ensuring quality information for patients.

* : Chi-Square test

p *values* in *bold* are *statistically significant*

According to the findings of this study, Gemini was found to be the easiest to read, and Perplexity was found to be the best in quality and reliability. All three AI models have a higher readability level than the recommended average readability level.

## Discussion

In this study, it was determined that the answers given by Perplexity, ChatGPT and Gemini AI chatbots to frequently asked questions regarding AS had a reading level above the 6th-grade level recommended by the US Department of Health and Human Services and the National Institutes of Health. A comprehensive evaluation has been conducted on the accuracy, reliability, and comprehensibility of AS-related information produced by these AI tools. Our study makes a significant contribution to the literature as one of the first comprehensive evaluations of answers to frequently asked questions about AS generated by popular large language models.

Health literacy is defined as the degree to which an individual can access, process, and comprehend basic health information and services, and therefore participate in health-related decisions [21]. Digital health literacy is defined as the ability to evaluate health information received from electronic sources and apply the obtained information to address or solve a health-related problem and is therefore known as an important component of health literacy. However, the use of medical terminology and jargon in the texts and dense paragraphs makes the texts difficult to understand and poses obstacle for those with limited health literacy [48]. It is stated that people with poor eHealth literacy tend to be significantly older and have more chronic health problems [49]. Providing internet and device access to individuals trying to access health-related online information, and ensuring that the information provided is at the recommended readability level, will help remove barriers to health literacy [48].

In the literature, studies investigating online information about AS have shown that texts have more difficult readability levels than recommended. In a study examining online information on publicly accessible websites about AS, the authors found that text readability levels were 4.1 ± 2.12 (mean ± SD) grade levels higher than the recommended 6th grade level [50]. In another study, the readability of written patient information and consent documents presented to patients in 24 rheumatological studies in the literature was evaluated and they found that they generally had a higher readability level than recommended for health literature [51]. In another study in which 200 websites were examined, it was determined that 46% of AS-related websites were high-quality, that sites sourced from scientific journals and news provided high quality information, and commercial websites provided low quality information. The authors stated that websites with poor readability provided high quality information [52]. AI is often mentioned as a potential solution to the problems facing healthcare today. It is suggested that AI has the potential to “give the gift of time” by allowing doctors and patients to use time more effectively in care [53].

For this reason, our study aimed to fill an important gap in the readability, reliability and quality of the answers given by AI-based chatbots, not online websites, to questions about a chronic disease such as AS.

To our knowledge, no studies have been conducted to comparatively examine the readability, quality, and reliability of responses from AI chatbots such as Gemini, ChatGPT, and Perplexity on the topic of AS. In a the study in which ChatGPT chatbots were examined to 60 questions asked in Spanish about some chronic diseases such as Ssystemic lupus erythematosus and Rheumatoid Arthritis, they determined the readability level of the answers obtained as “moderately difficult”. The authors reported that such practices tend to produce more successful results in the language in which they are trained, and therefore future studies comparing the responses given in Spanish and English may shed light on the subject [54].

In a study in which the answers to 30 rheumatological questions asked to the AI chatbot called Microsoft Bing Chat were evaluated, rheumatologists found that these answers were of low quality and poor readability. The authors stated that rheumatology patients should be careful when using these AI tools when answering their medical questions [55]. In the study, which examined the answers given by five AI chatbots, namely Gemini, Microsoft Copilot, PiAI, ChatGPT and ChatSpot, to 45 questions created based on the recommendations in the guidelines on psoriasis, cardiovascular health and oncology, the readability levels of the answers were determined as “advanced and academically based level”. The authors reported that these answers may be difficult to understand for individuals with less than a university-level education , and that the answers provided by their chatbots vary in terms of sentence length, readability, consistency, and accuracy [56].

There are studies in the literature that evaluate the answers given by AI not only in rheumatological diseases but also in other medical subjects, such as palliative care, subdural hematoma, or low back pain. In these studies, it has been clearly stated that AI chatbots can offer the opportunities to improve health outcomes and patient satisfaction, but the high level of readability prevents this situation [13,20,21].

In parallel with other studies in the literature, our research, it was determined that the readability level of the content about AS produced by AI models such as Gemini, Perplexity and ChatGPT was above the sixth- grade level recommended by the NIH and AMA. The easiest readability was found in Gemini, and the most difficult was found in Perplexity. The presentation of more readable text by AI will allow doctors and patients to better collaborate with technology, maintaining a human-centered approach to care. Not only this, it has been reported that the use of AI in rheumatoid arthritis has a promising role in early diagnosis and treatment development. It is reported that incorporating appropriate algorithms, machine and/or deep learning algorithms into real-world environments can increase the utility of future next-generation AI applications [15]. The results of our study also emphasize that AI applications that will provide appropriate readability and high-quality reliable information in the future may positively affect public health.

In this study, not only the readability but also the medical accuracy and reliability of the information provided by AI chatbots about AS were carefully examined. In this way, iwe attempted to reveal how reliable a source of information these tools provide in the field of health. In the study where the answers given by Microsoft Copilot with ChatGPT 4.0 to 15 frequently asked questions about pediatric familial Mediterranean fever were evaluated, it was determined that there were important inaccuracies and omissions with poor reliability. The authors stated that despite the valuable functions and potential of AI models, the lack of validated methods to determine the reliability and accuracy of the information they provide is still prevalent [57]. In another study examining the the responses provided by ChatGPT3.5 about complementary and alternative medicine in rheumatology, it was reported that the responses lacked a scientific basis. [58]. Different from the results of current study, another study examining AI models on magnetic resonance imaging in rheumatology found that AI shows significant promise with potential uses in disease diagnosis, classification and management in MRI in rheumatology, and that it is often comparable to or compared to expert radiologists and rheumatologists in SpA management aspects. They reported that they were able to achieve performance that exceeded them. However, the authors reported that there are still challenges to be overcome, such as the need for large, high-quality datasets and the integration of AI into clinical workflows [59]. There are studies in the literature showing that AI chatbots provide reliable information to questions about rheumatological diseases [54,56]. In our research, it was observed that the Perplexity language model, like other studies in the literature, achieved significantly higher scores than other models on the quality and reliability scales [12,21]. The date, author and source information added to the answers of the Perplexity model caused the model to differ from other models in terms of information reliability and quality. These findings show that the advantages offered by the Perplexity model should be taken into account in the development of AI-supported healthcare services.

However, during the presentation of AI-based health information, it should not be forgotten that the medical decisions that patients can make can never replace face-to-face medical consultation due to weak points regarding security, lack of transparency in AI algorithms, and the possibility of fabricated information in the content created by AI. In addition, due to the responses given by chatbots, possible misinformation or overconfidence in patients may cause life-threatening situations and affect public health.

### Limitations

We can list the limitations of our study as follows. This study we conducted based on the first 25 keywords offered by Google to reach the most up-to-date information about AS can be made more comprehensive by supporting it with a larger keyword pool. In addition, it should not be ignored that the word group axial apondyloarthritis, which has a similar meaning, may have been used by users in the search engine instead of AS, and this may change the research results. Future studies conducted with the keyword axial spondyloarthritis will shed light on the quality and safety of responses given by AI chatbots in a broader terminology, including nonradiographic axial spondyloarthritis.

The scope of the study is limited to keywords specific to the English-language. This prevents more comprehensive inferences about the impact of language. Although AI applications have the capacity to create and process text in multiple languages, their performance is often better in the language they were trained in. Therefore, the accuracy of answers given to questions in English may differ from the accuracy of answers in other languages. Not only this, but due to the use of European languages and Roman alphabets, it is clear that the results will differ in different language structures and alphabets [54]. The issue can be clarified in the future with studies conducted with different language groups or comparing English with a different language. Comparisons with different and more diverse AI models than those we used in our study will allow us to better understand the limits and potential of this technology. Our analysis is based on a static dataset for October 2024. This situation limits the ability to capture change over time and current trends. Considering that new models are released every day and new technologies and new features are included, there is no small possibility that we will receive very advanced reliable and quality information in a different time period in the future. In future studies, the generalizability of the results can be increased by considering more languages and dynamic datasets.

### Study strengths

Our study is the first to evaluate not only the readability of AI chatbots regarding AS, but also quality and reliability metrics such as information accuracy, consistency, and referencing. Unlike other studies, by comparing multiple popular AI chatbots, it more clearly reveals the potential and limits of this technology in healthcare. These results may contribute to the development of more reliable and effective AI-based healthcare services by providing a roadmap for future studies.

## Conclusion

AI-powered chatbots (ChatGPT, Perplexity, Gemini) are becoming increasingly capable of providing information on medical topics such as AS. However, there are still question marks about the readability and reliability of the content produced by these chatbots. In our study, it was determined that the AS-related information produced by these chatbots was of a complexity above the sixth-grade grade level, which is considered to be understandable by a large part of the general population. According to the results of this study, it can be emphasized that although AI chatbots provide useful information for patients and healthcare professionals on medical issues in daily practice, users should be alert against the possible danger of misinformation.

Future advances in AI may improve patient care through improved diagnostic accuracy and personalized treatment strategies, but healthcare providers, AI developers, and policymakers have a role to play to ensure this. Based on the results of this study; it can be interpreted that AI developers should establish the necessary infrastructure to provide accurate and readable information that will not endanger public health by new model AI technologies in the future, healthcare providers should be suspicious of the reliability of the information provided by AI, and policymakers should impose some penalties on AI applications that pose a threat to public health by providing misinformation. It is clear that in future studies, addressing user comprehension issues and new AI models to be produced in languages other than English can help in the development of AI and in reaching more people with more understandable and less erroneous information.
